# Supplementary material for: Deadenylation and decapping factors cooperatively stimulate biochemical activities of DEAD-box ATPase Dhh1
Source: Nucleic Acids Res. 2026 Sep 18;54(17):gkag908. doi: 10.1093/nar/gkag908 (PMC13586536; doi:10.1093/nar/gkag908)
Supplement: Supplementary file 1 [file gkag908_supplemental_file.pdf]

SUPPLEMENTARY INFORMATION

Deadenylation and Decapping Factors Cooperatively Stimulate Biochemical Activities of DEAD-Box ATPase Dhh1

Gabriel A. Braun,[1] Rakesh Kumar,[2] Alan G. Hinnebusch,[2] John D. Gross[1,*]

[1]Department of Pharmaceutical Chemistry, University of California, San Francisco, California, 94158, United States

[2]Division of Molecular and Cellular Biology, Eunice Kennedy Shriver National Institute of Child Health and Human Development, National Institutes of Health, Bethesda, Maryland, 20892, United States

*To whom correspondence should be addressed. Email: jdgross@cgl.ucsf.edu

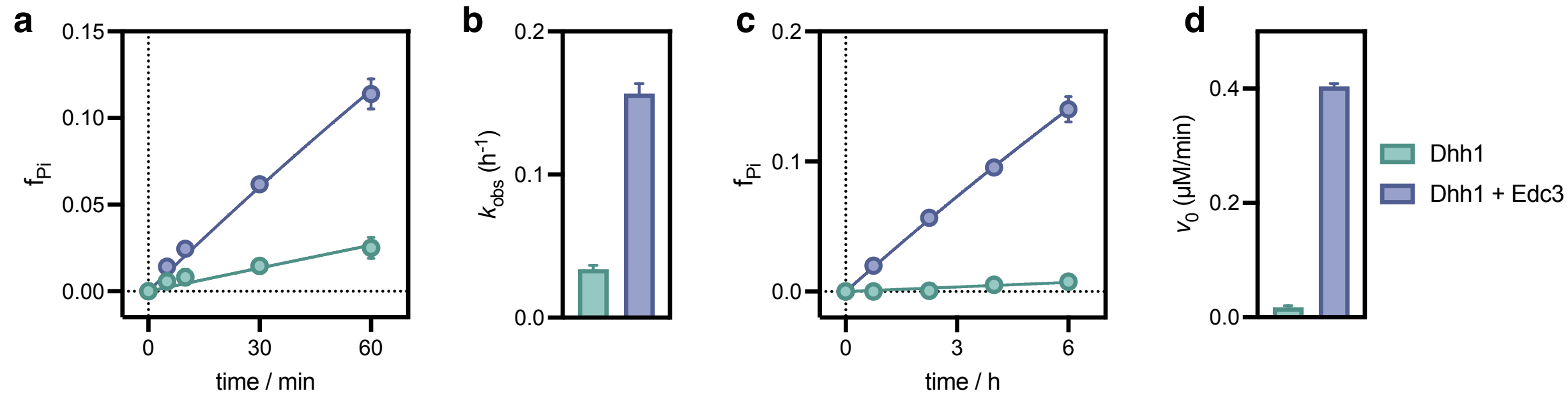

Figure S1. Edc3 stimulates Dhh1 ATPase under single- and multiple-turnover conditions ATPase activity of 5 *μ*M Dhh1 in the absence (green) and presence (blue) of 15 *μ*M Edc3 under **(a-b)** single-turnover and **(c-d)** multiple-turnover conditions. Total ATP concentration is 1 nM and 1 mM, respectively, and both conditions contain saturating amounts of U_{10} RNA. Data points in panels (a) and (c) show mean and standard deviation from n = 3 technical replicates; error bars in panels (b) and (d) show standard deviation of fitted rates. See **Tables S3** and **S4** for single- and multiple-turnover ATPase rates shown in panels (b) and (d).

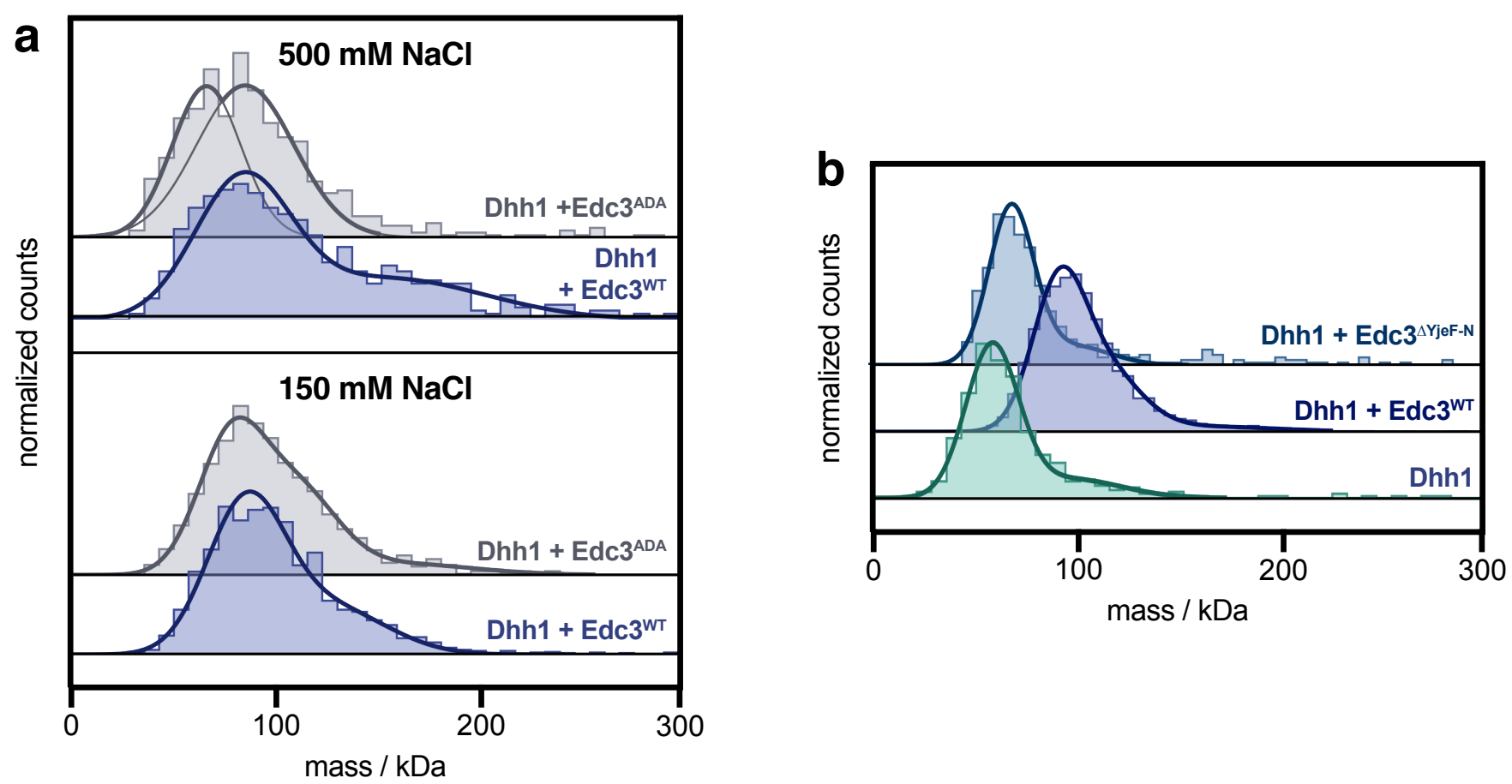

Figure S2. Edc3^{ADA} and Edc3$^{YjeF\text{-}N}$ form complexes with Dhh1

a. Mass photometry of binary mixtures of Dhh1 and either Edc3^{WT} (blue) or Edc3^{ADA} (grey), under standard salt (150 mM NaCl) and high salt (500 mM NaCl) conditions. **b.** Mass photometry of binary mixture of a binary mixture of Dhh1 and Edc3$^{\Delta YjeF\text{-}N}$ (light blue); Dhh1 alone (green) and Dhh1 with Edc3^{WT} (dark blue) are included for reference. (Monomeric molecular weights: Dhh1: 55 kDa; Edc3^{WT} and Edc3^{ADA}: 49 kDa; Edc3$^{\Delta YjeF\text{-}N}$: 21 kDa.) See **Table S9** for experimental and theoretical masses for complexes shown in both panels.

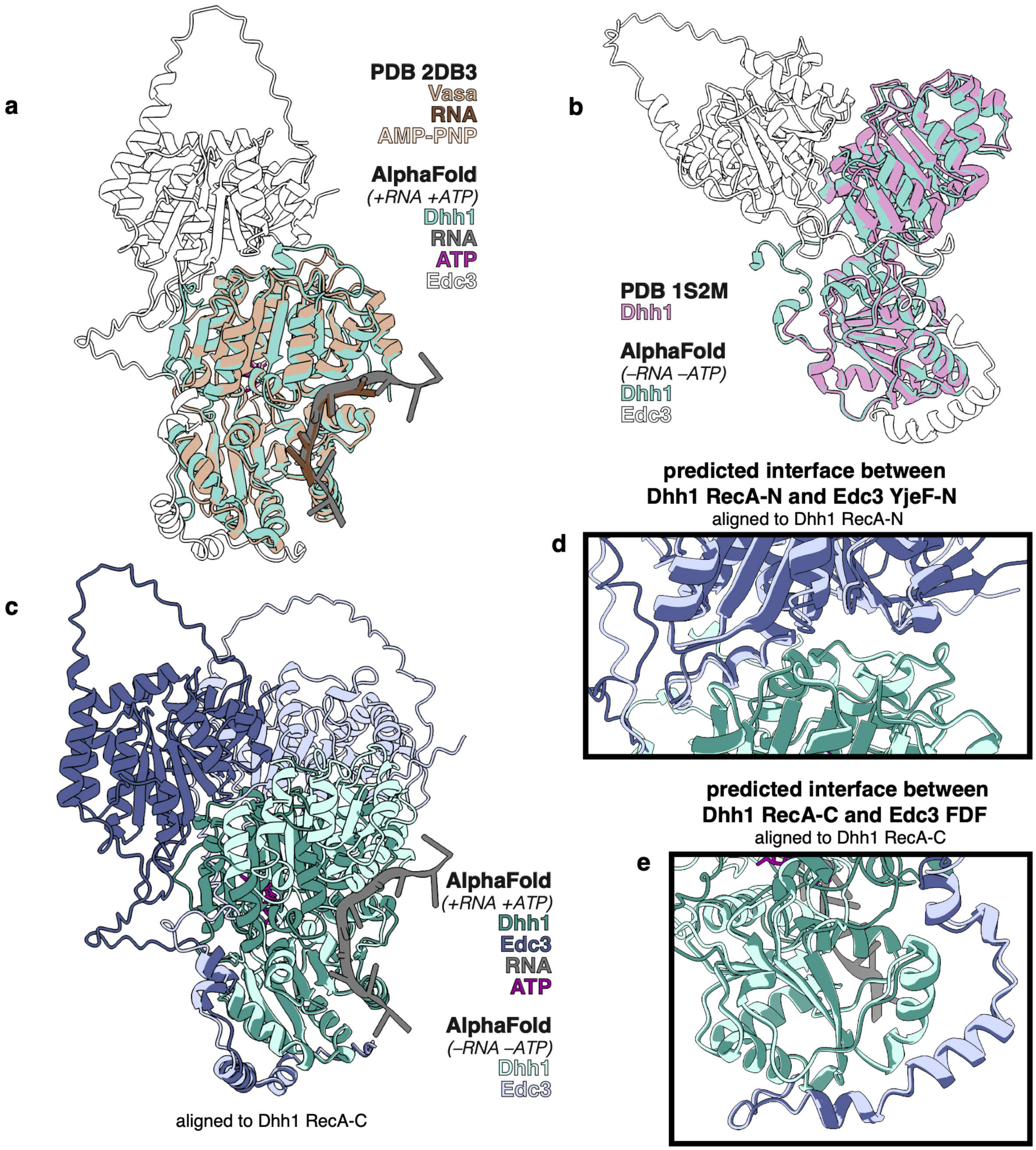

Figure S3. Dhh1-Edc3 structural predictions in the presence and absence of RNA and ATP
a. Predicted Dhh1-Edc3 dimer in the presence of RNA and ATP, compared to experimental structure of *Drosophila* DBP Vasa in active state (PDB: 2DB3). **b.** Predicted Dhh1-Edc3 dimer in the absence of RNA and ATP, compared to experimental structure of Dhh1 in inactive state (PDB: 1S2M). **c.** Prediction of Dhh1/Edc3 heterodimer in the presence (dark colors) and absence (light colors) of RNA and ATP. **d-e.** Isolated interaction interfaces are similar regardless the presence of RNA and ATP, including the interaction between **(d)** Edc3 YjeF-N domain and Dhh1 RecA-N and **(e)** Edc3 FDF region and Dhh1 RecA-C. Coloring is the same as panel (c). For all predicted structures, the Dhh1 N- and C-terminal IDRs and the Edc3 Lsm domain are hidden for clarity.

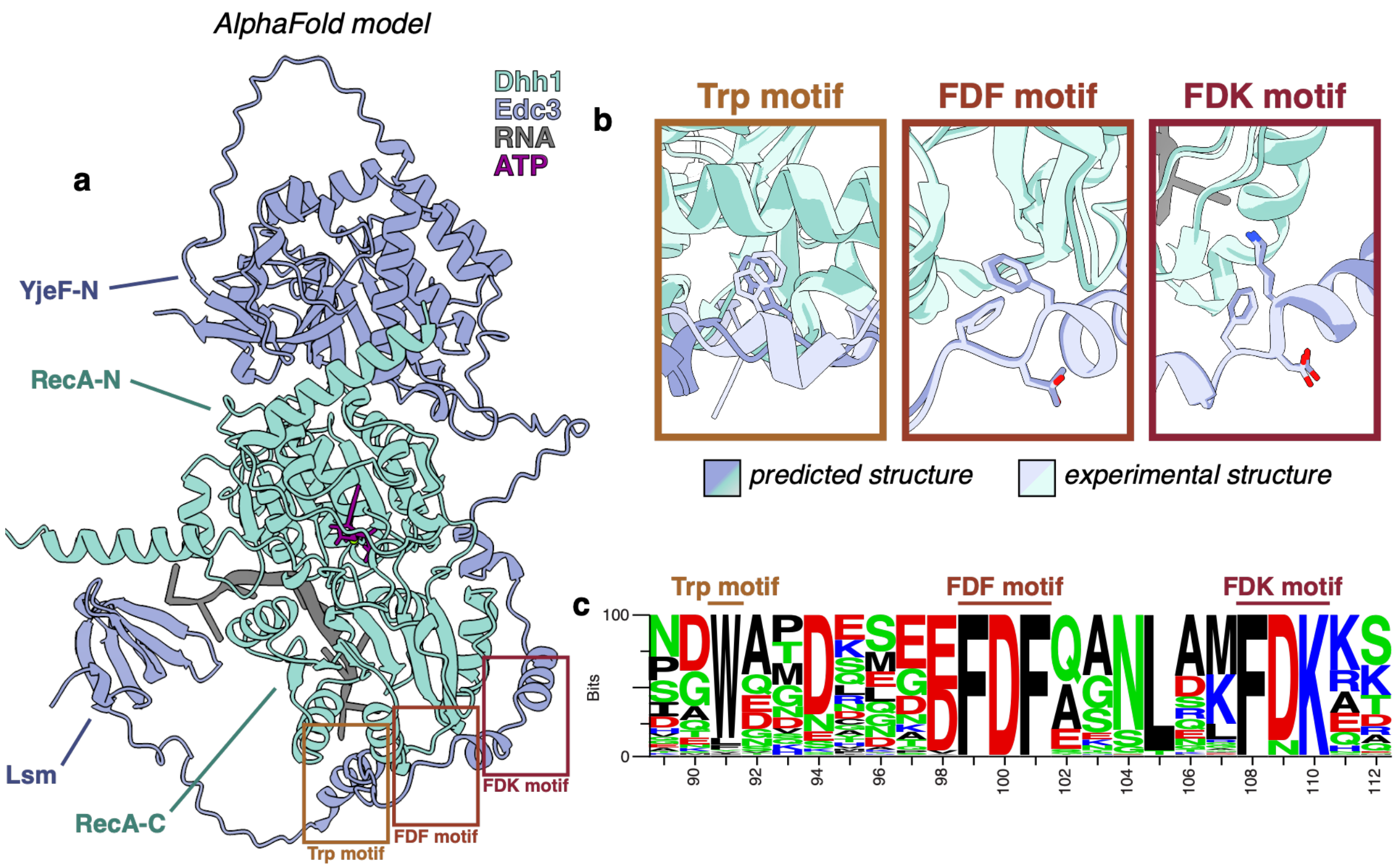

Figure S4. Comparison of AlphaFold prediction with experimental Dhh1-Edc3 structures
a. Predicted structure of Dhh1-Edc3 heterodimer, as shown in Fig. 5, with the locations of the conserved Trp, FDF, and FDK motifs indicated in boxes. **b.** Comparison of predicted interaction sites with experimental structures; for all, predicted structure is shown in dark blue and green and experimental structures are shown in light blue and green. (PDB: Trp motif: 4BRU; FDF & FDK motifs: 2WAX) **c.** Logo plot showing sequence conservation in the known Dhh1-interacting region of Edc3, with known interaction motifs marked.

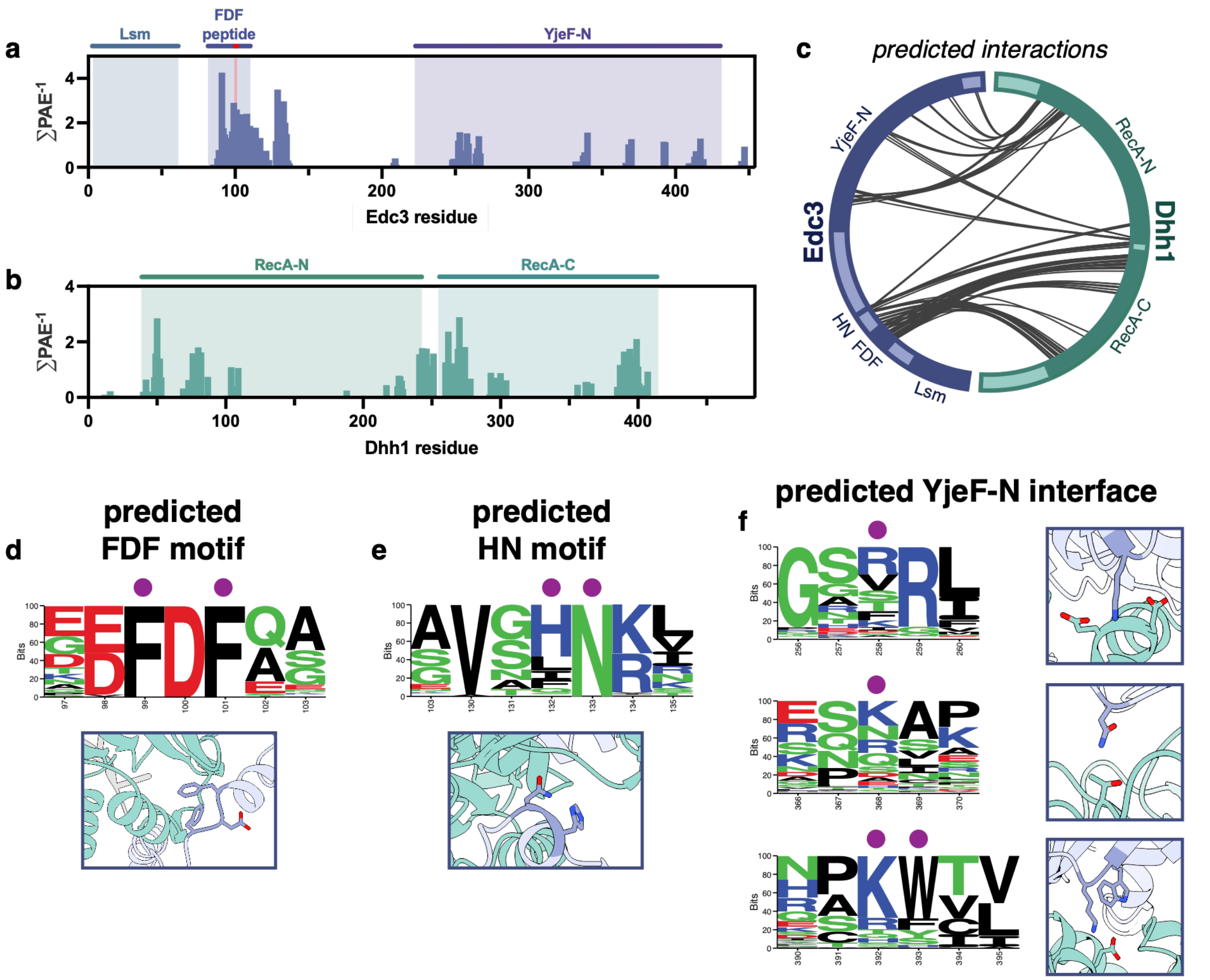

Figure S5. Predicted Dhh1-Edc3 heterodimer includes new interaction interfaces

a-b. Sites of predicted interaction between Dhh1 and Edc3 across the sequence of **(a)** Edc3 and **(b)** Dhh1, quantified as the per-residue sum of PAE^{-1} for interactions under 4 Å. For each protein, domains are annotated; the FDF peptide corresponds to $Edc3^{FDF}$ construct tested experimentally (see Fig. 2e-g). **c.** Contact map of predicted Dhh1-Edc3 interactions. Lines show resides within 4 Å with PAE values < 4 Å. **d-f.** Sequence conservation and predicted interaction sites for **(d)** FDF motif, **(e)** HN motif, and **(f)** YjeF-N interface. Mutated residues are indicated with purple dots.

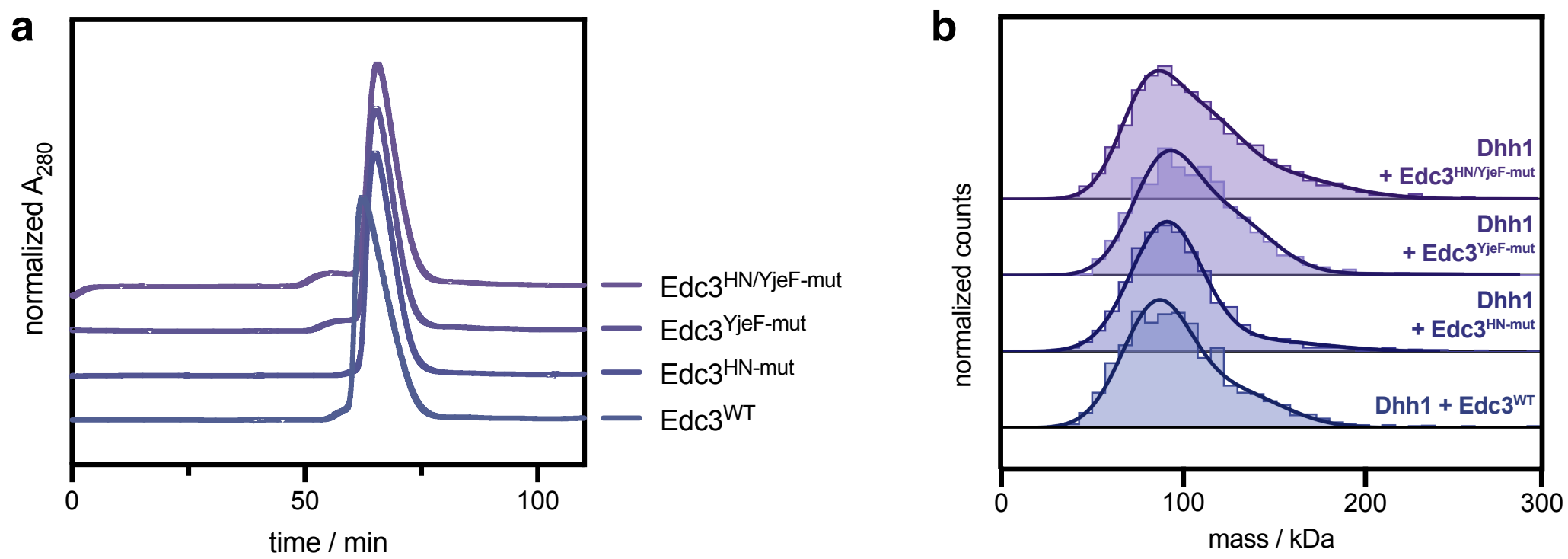

Figure S6. Edc3 mutations do not compromise solubility or interaction with Dhh1
a. Size-exclusion chromatography of Edc3^{WT}, Edc3$^{HN\text{-}mut}$, Edc3$^{YjeF\text{-}mut}$, and Edc3$^{HN/YjeF\text{-}mut}$. **b.** Mass photometry of binary mixtures of Dhh1 with Edc3^{WT}, Edc3$^{HN\text{-}mut}$, Edc3$^{YjeF\text{-}mut}$, and Edc3$^{HN/YjeF\text{-}mut}$, and Edc3^{ADA}. (Monomeric molecular weights: Dhh1: 55 kDa; all Edc3 variants: 49 kDa.) See **Table S9** for experimental and theoretical masses for complexes shown in panel (b).

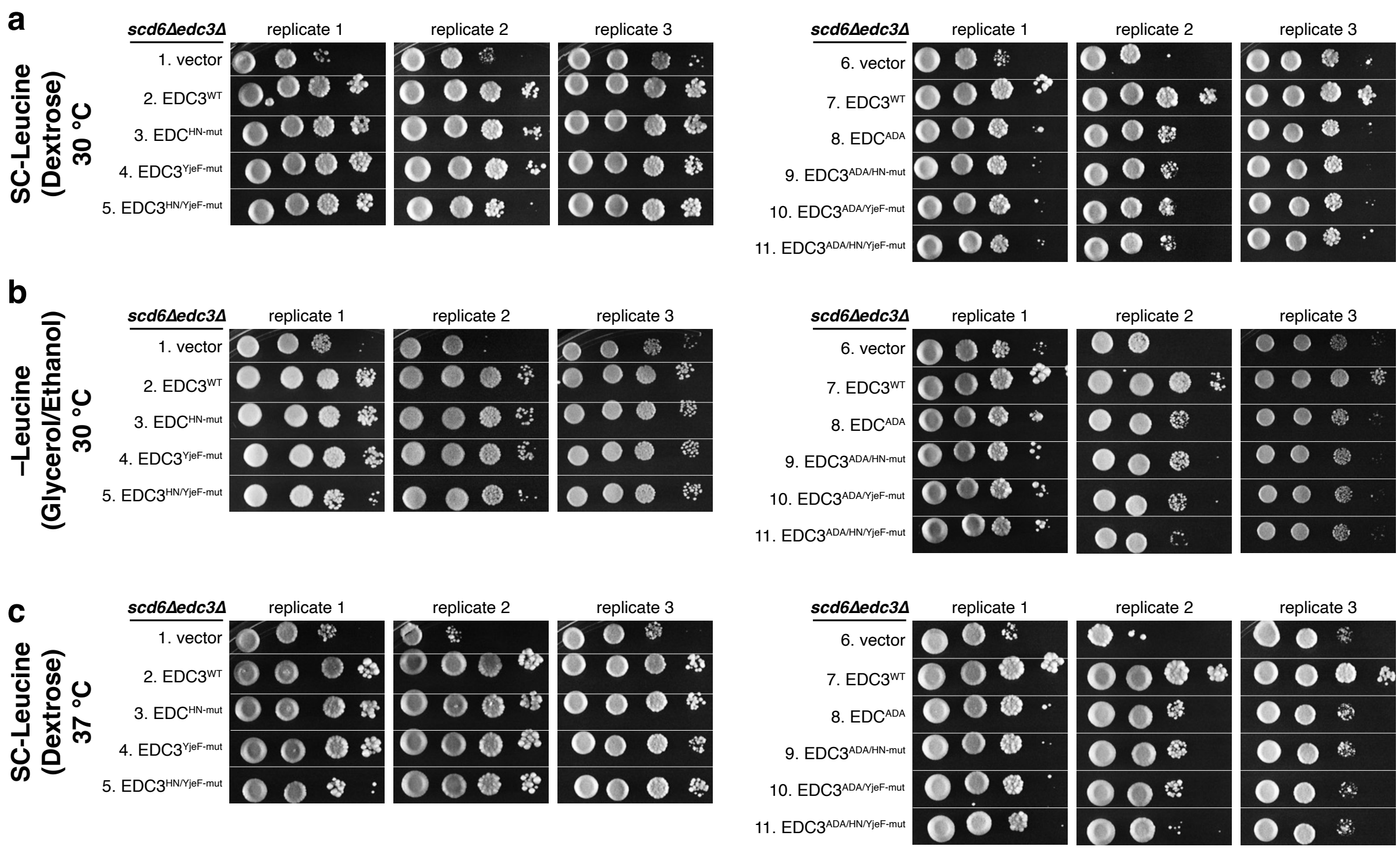

Figure S7. Biological replicates for yeast growth from Figure 3g

Cell growth of transformants of a *scd6ΔedcΔ S. cerevisiae* strain harboring either wild-type or mutant *EDC3* alleles, all tagged with the *myc13* epitope, on single-copy plasmids. Shown are results from three independent transformations. Cells were grown on **(a)** plates including leucine at 30 °C, **(b)** plates lacking leucine at 30 °C, and **(c)** plates including leucine at 37 °C. Spots represent 10-fold serial dilutions. Data from replicate 1 is shown in **Figure 3g**.

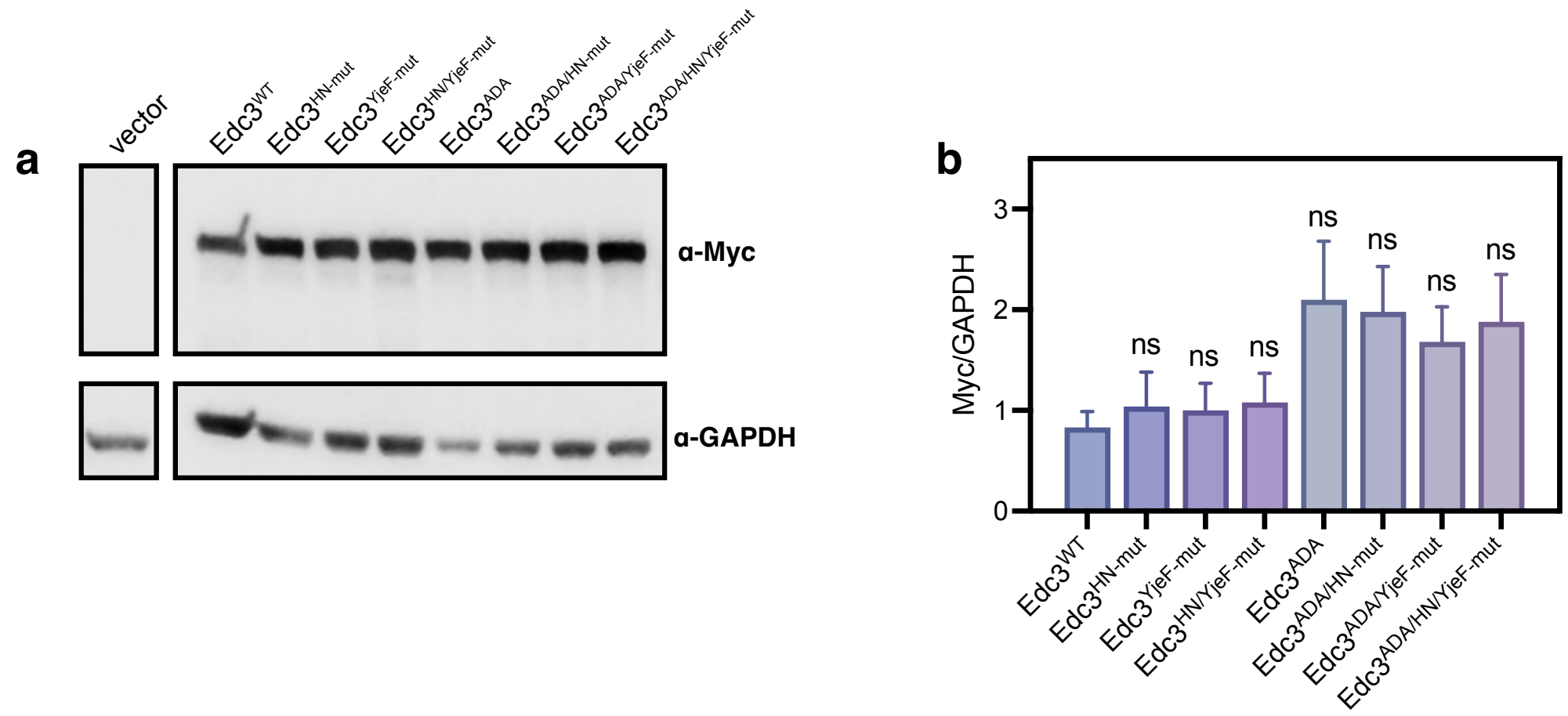

Figure S8. Expression of wild-type and mutant *EDC3* in *S. cerevisiae*

a. Western blot analysis of the expression if myc13-tagged wild-type and mutant Edc3 proteins in the same transformants of the *scd6Δedc3Δ* strain analyzed for cell growth **(Fig. 3g** and **Fig. S7)**, with GAPDH used as a loading control. **b.** Quantified ratio of Myc-tagged Edc3 to GAPDH from blots of n = 3 biological replicates, with error bars showing standard deviation. (Statistical analysis: one-way ANOVA with Dunnett's multiple comparison test versus Edc3^{WT}, $n = 3$.) See **Table S12** for data shown in panel (b).

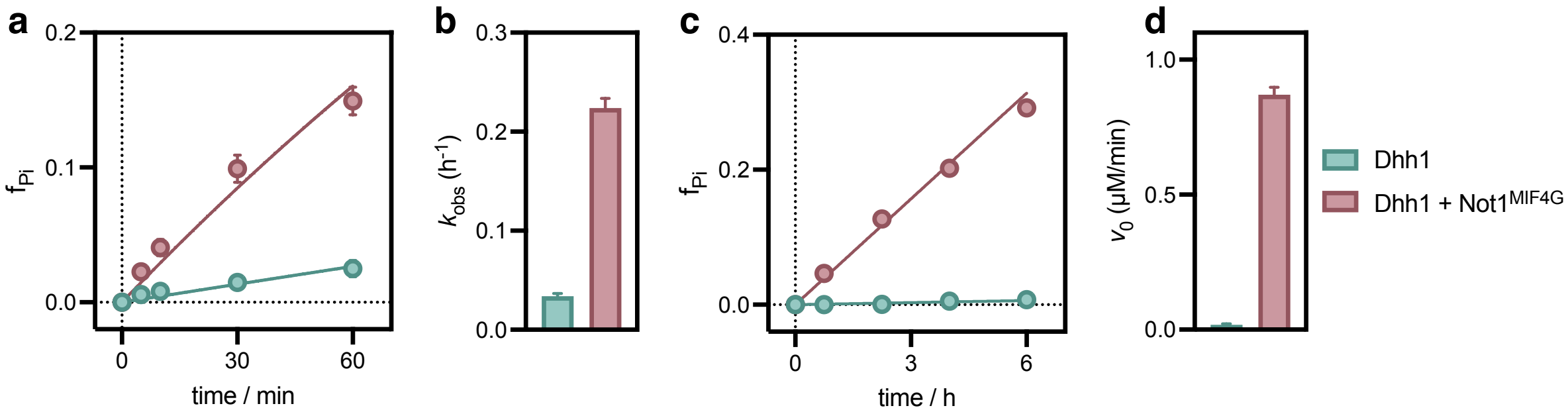

Figure S9. Not1^{MIF4G} stimulates Dhh1 ATPase activity

ATPase activity of 5 μM Dhh1 in the absence (green) and presence (red) of 15 μM Not1^{MIF4G} under **(a-b)** single-turnover and **(c-d)** multiple-turnover conditions. Total ATP concentration is 1 nM and 1 mM, respectively, and both conditions contain saturating amounts of U_{10} RNA. Data points in panels (a) and (c) show mean and standard deviation of n = 3 technical replicates; error bars in panels (b) and (d) show standard deviation of fitted rates. See **Tables S3** and **S4** for single- and multiple-turnover ATPase rates shown in panels (b) and (d).

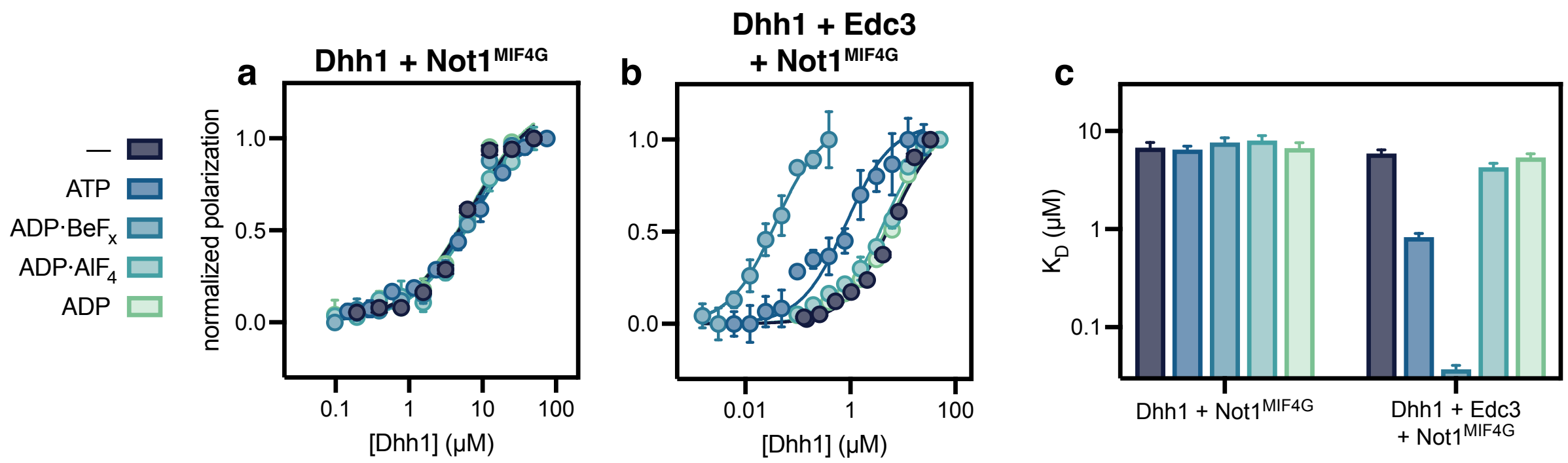

Figure S10. Not1^{MIF4G} does not affect affinity of Dhh1 for RNA in binary and ternary mixtures Fluorescence polarization of Dhh1 binding to a fluorescein-labeled U_{20} RNA in the presence of saturating amounts of **(a)** Not1^{MIF4G} or **(b)** both Edc3 and Not1^{MIF4G} in the absence of nucleotide or with various nucleotides and nucleotide analogs. Data points show mean and standard deviation of n = 3 technical replicates. **c.** Fitted K_D values for data shown in panels (a-b), with error bars showing standard deviation. See **Table S7** for K_D values shown in panel (c).

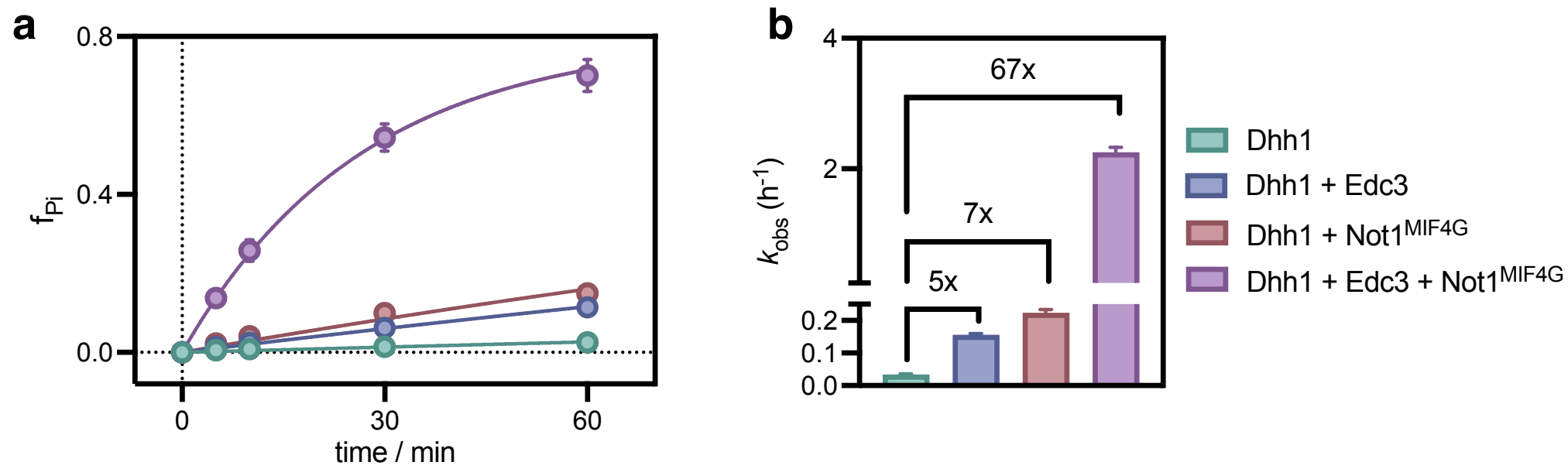

Figure S11. Edc3 and Not1^{MIF4G} cooperatively stimulate Dhh1 ATPase under single-turnover conditions

ATPase activity of 5 μM Dhh1 alone (green) and in the presence of 15 μM Edc3 (blue), Not1^{MIF4G} (red), or both Edc3 and Not1^{MIF4G} (pink) under single-turnover conditions. Reactions contain saturating amounts of U_{10} RNA. Data points in panel (a) show mean and standard deviation of n = 3 technical replicates; error bars in panel (b) show standard deviation of fitted rates. See **Table S3** for ATPase rates shown in panel (b).

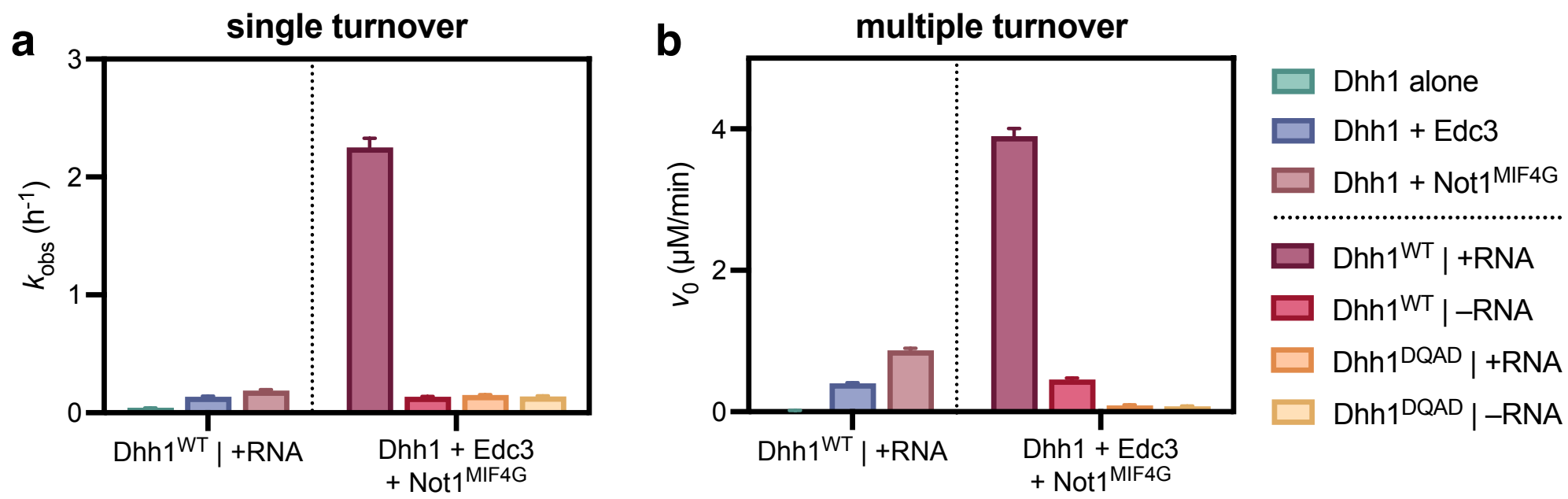

Figure S12. Stimulation of Dhh1 by Not1 in ternary mixtures requires RNA and Walker B glutamate

Activity of either Dhh1^{WT} or Dhh1^{DQAD} in the presence of both Edc3 and Not1^{MIF4G} in both the presence and absence of RNA under **(a)** single-turnover and **(b)** multiple-turnover conditions. Total ATP concentration is 1 nM and 1 mM, respectively. Included for reference in each condition is the activity of Dhh1^{WT} in the presence of RNA either alone (green), with Edc3 (blue), or with Not1^{MIF4G} (red). Bars show fitted rate with standard deviation from n = 3 technical replicates. See **Tables S3** and **S10** for single-turnover rates shown in panel (a) and **Tables S4** and **S11** for multiple-turnover rates shown in panel (b).

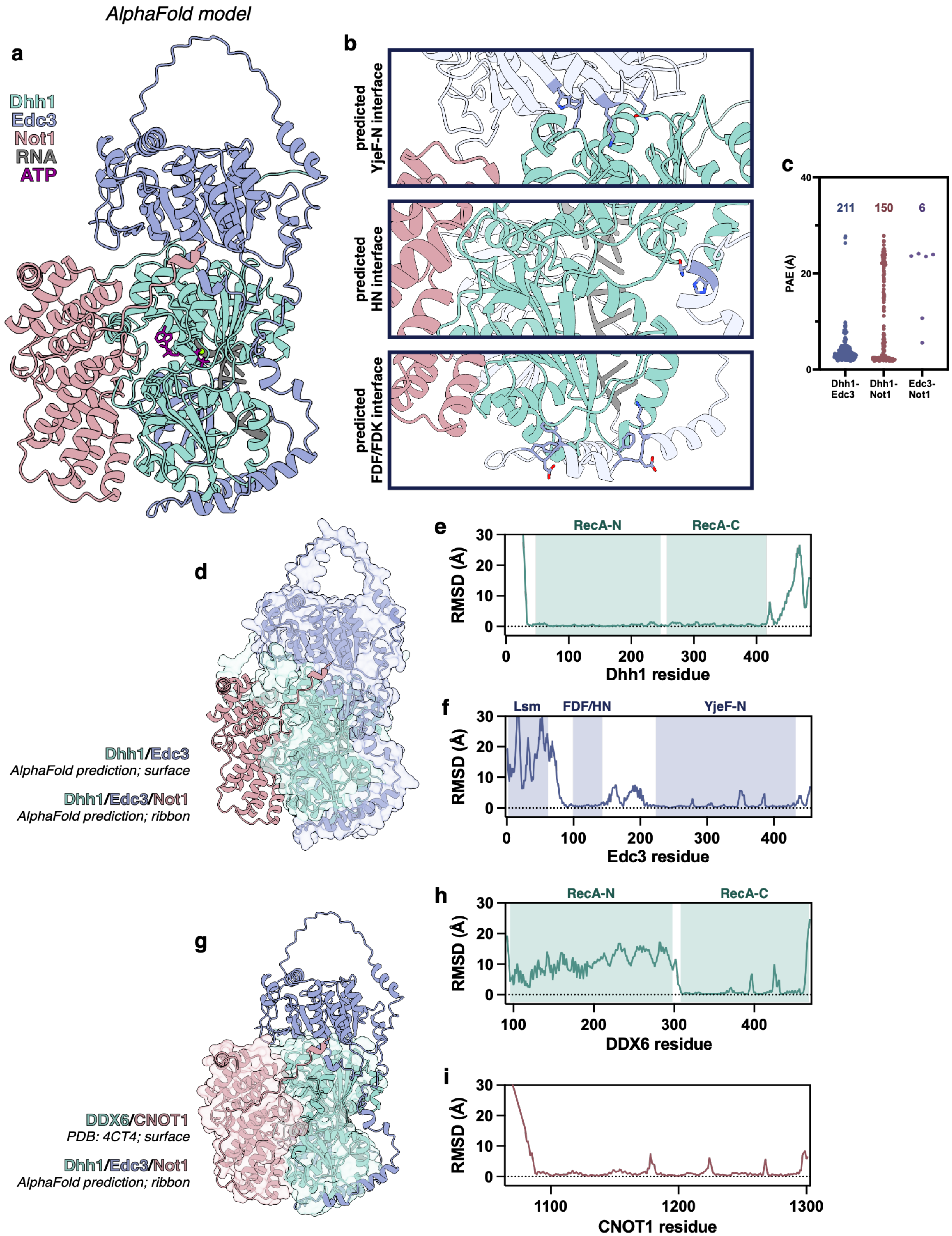

Figure S13. Predicted structure of a heterotrimeric Dhh1-Edc3-Not1^{MIF4G} complex

a. Predicted structure between Dhh1 (green), Edc3 (blue), and Not1^{MIF4G} (red), in the presence of U_{10} RNA (grey) and ATP (purple). **b.** Close-up views of Dhh1-Edc3 interfaces in predicted

Dhh1/Edc3/Not1^{MIF4G} heterotrimer shown in panel (a), demonstrating that known interfaces are unimpeded by the presence of Not1^{MIF4G}. Highlighted are Edc3 residues mutated at YjeF-N and HN interfaces to make Edc3$^{YjeF\text{-}mut}$ and Edc3$^{-HN\text{-}mut}$, as well as FDF and FDK motifs. **c.** Predicted aligned error (PAE) for intermolecular residue pairs within 5 Å, with the number of residue pairs shown for each pair of proteins. Low PAE indicates higher model confidence.
d. Comparison of predicted Dhh1/Edc3/Not1^{MIF4G} heterotrimer (shown in ribbons) with predicted Dhh1/Edc3 heterodimer (shown as transparent surface). **e-f.** RMSD between structures shown in panel (d) for **(e)** Dhh1 (green) and **(f)** Edc3 (blue). **g.** Comparison of predicted Dhh1/Edc3/Not1^{MIF4G} heterotrimer (shown in ribbons) with DDX6/CNOT1^{MIF4G} crystal structure (PDB: 4CT4, shown as transparent surface). **h-i.** RMSD between structures shown in panel (g) for **(h)** DDX6 (green) and **(i)** CNOT1 (red). (The interdomain orientation of Dhh1 differs between the active RNA-bound predicted model and the non-RNA-bound crystal structure, leading to the uniformly high RMSD for the DDX6 RecA-N.)

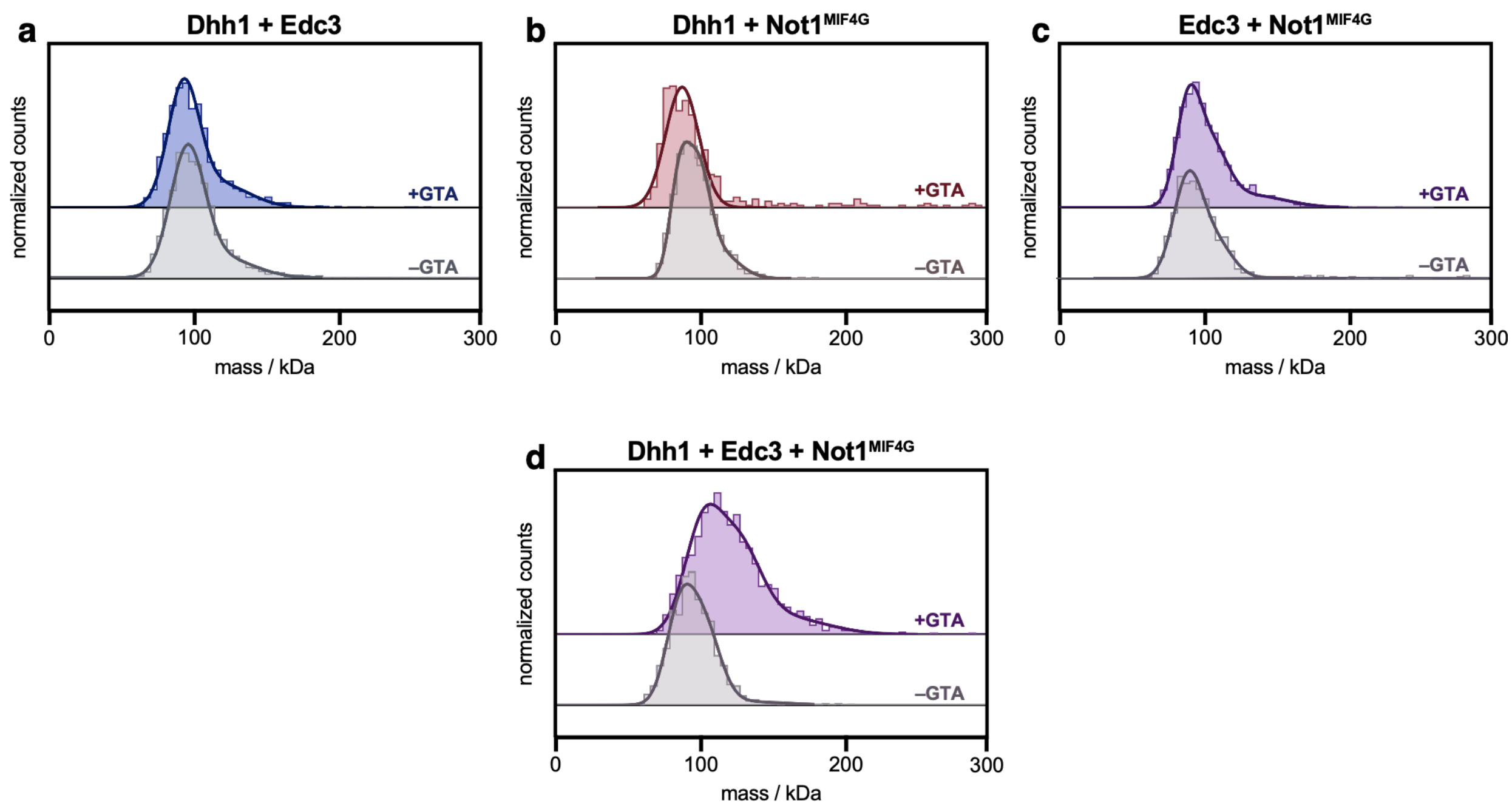

Figure S14. Crosslinking mass photometry of binary and ternary mixtures

Mass photometry of different combinations of Dhh1, Edc3, and Not1^{MIF4G} with and without glutaraldehyde crosslinking. **a.** Binary mixture of Dhh1 and Edc3. **b.** Binary mixture of Dhh1 and Not1^{MIF4G}. **c.** Binary mixture of Edc3 and Not1^{MIF4G}. (The peak observed for this binary mixture likely corresponds to an Edc3 homodimer, with unbound Not1^{MIF4G} below the detection limit of the technique.) **d.** Ternary mixture of Dhh1, Edc3, and Not1^{MIF4G}. See **Table S9** for experimental and theoretical masses for all complexes.

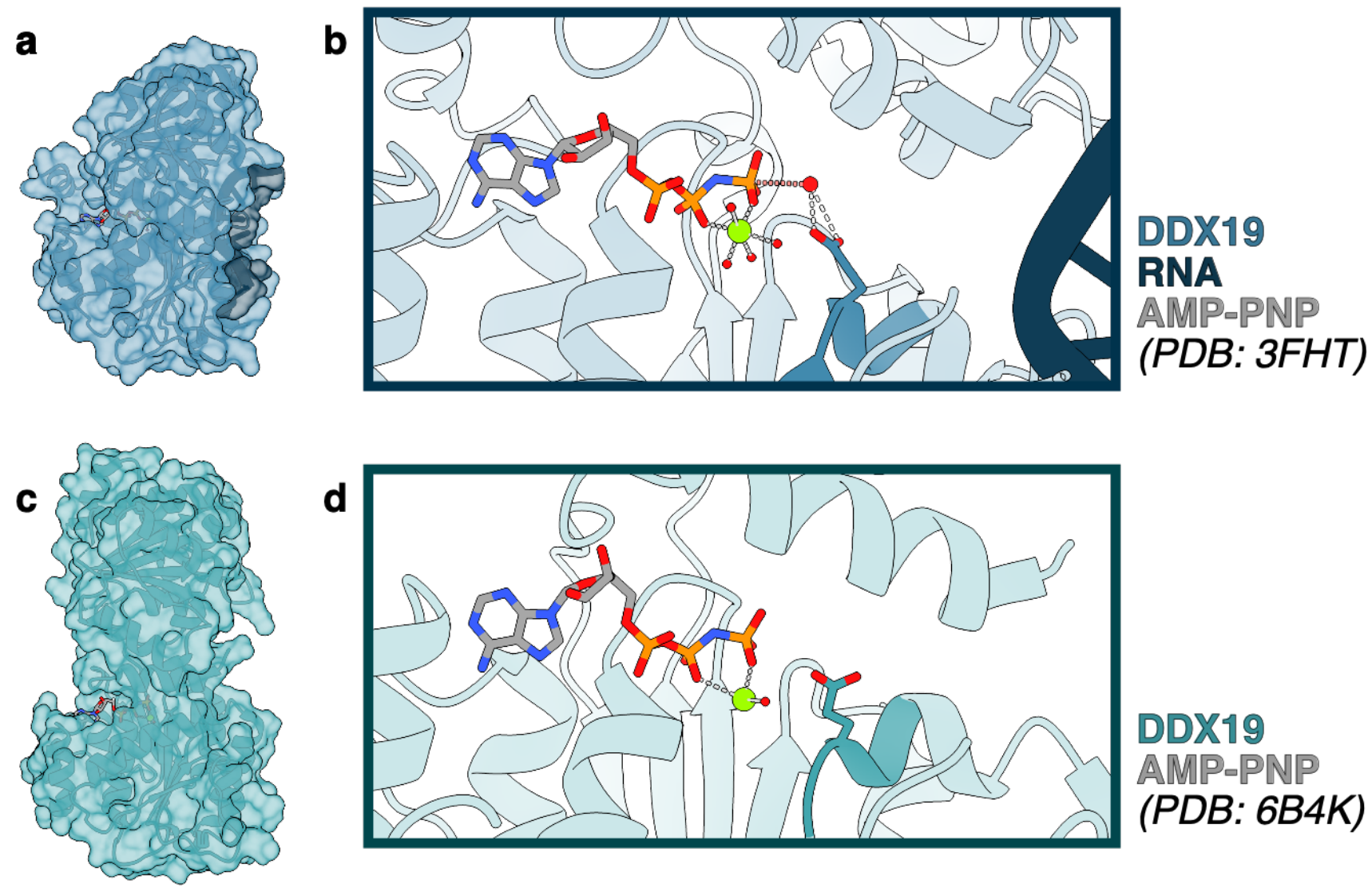

Figure S15. Orientation of DEAD-box catalytic glutamate is sensitive to RNA

Crystal structures of the human DBP DDX19 bound to AMP-PNP in the **(a-b)** presence and **(c-d)** absence of RNA. **a.** In the presence of RNA, DDX19 adopts a compacted tertiary structure. (PDB: 3FHT) **b.** The active site of DDX19 in the presence of RNA, with the Walker B (DEAD) motif highlighted. The glutamate of the Walker B motif forms hydrogen bonds to a water molecule positioned for hydrolysis, 3.1 Å from the AMP-PNP γ-phosphate. **c.** In the absence of RNA, DDX19 adopts an extended, open conformation. (PDB: 6B4K) **d.** The DDX19 active site in the absence of RNA. The Walker B motif is oriented away from the AMP-PNP, with no potential nucleophilic water molecules within 4 Å of the γ-phosphate.

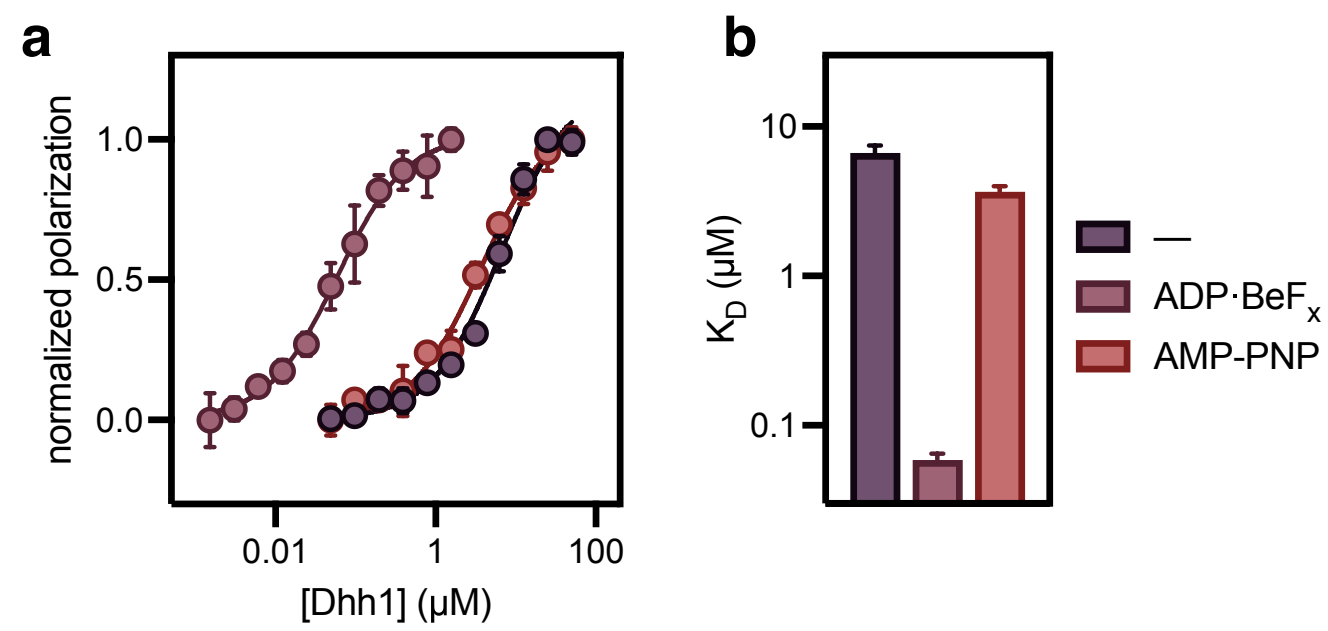

Figure S16. Edc3 does not increase Dhh1 affinity for RNA in the presence of AMP-PNP
a. Fluorescence polarization for Dhh1 binding to a fluorescein-labeled U_{20} RNA in the presence of saturating amounts of Edc3 in the absence of nucleotide or with either ADP-BeF_x or AMP-PNP. Data points show mean and standard deviation of n = 3 technical replicates. **b.** Fitted K_D values for data shown in previous panel, with error bars showing standard deviation. Data in the absence of nucleotide and with ADP-BeF_x are repeated from Figure 2 to allow for comparison. See **Table S8** for K_D values shown in panel (b).

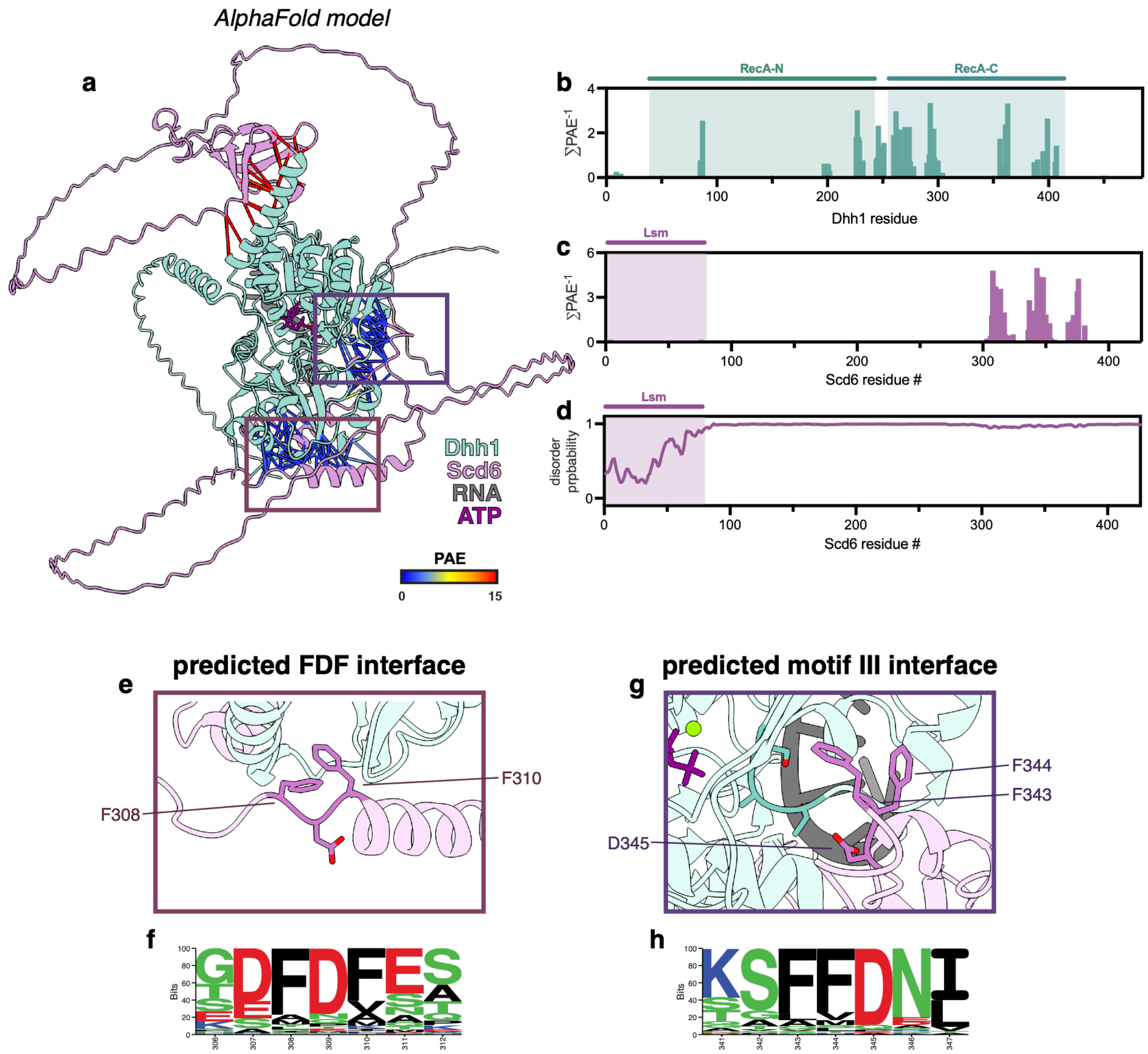

Figure S17. Predicted Dhh1-Scd6 heterodimer includes contact analogous to HN motif
a. AlphaFold 3 prediction of heterodimer between Dhh1 (green) and Scd6 (pink), in the presence of RNA (grey) and ATP (purple). Residues with predicted interprotein distances under 4 Å are shown with connecting lines, colored by Predicted Aligned Error (PAE). Boxed regions show predicted interfaces shown in greater detail in panels (e) and (g). **b-c.** Sites of predicted interaction between Dhh1 and Scd6 across the sequence of **(a)** Dhh1 and **(b)** Scd6, quantified as the per-residue sum of PAE^{-1} for interactions under 4 Å. For each protein, structured domains are annotated. **d.** AIUPred disorder prediction for Scd6 (1). **e-f.** Scd6 FDF motif, showing **(e)** the predicted interaction with Dhh1 and **(f)** sequence conservation. **g-h.** Scd6 motif that interacts at similar interface to Edc3 HN motif (see Figure 3), showing **(g)** the predicted interaction with Dhh1 (Motif III of is Dhh1 colored in dark green), and **(h)** sequence conservation.

Table S1. Constructs used for recombinant protein expression

protein construct	amino acid boundaries	mutations	purification tag	vector	organism
**Dhh1**	1-485	—	6xHis-TEV	pETDuet	*S. pombe*
**Dhh1^{DQAD}**	1-485	E194Q	6xHis-TEV	pETDuet	*S. pombe*
**Edc3**	1-454	—	6xHis-TEV	pET-30b	*S. pombe*
**Edc3^{ADA}**	1-454	F99A, F101A	6xHis-TEV	pET-30b	*S. pombe*
**Edc3$^{\Delta Lsm}$**	62-454	—	6xHis-TEV	pET-30b	*S. pombe*
**Edc3$^{\Delta YjeF\text{-}N}$**	1-192	—	6xHis-TEV	pET-30b	*S. pombe*
**Edc3^{FDF}**	81-110	—	—	pET-30b	*S. pombe*
**Edc3$^{HN\text{-}mut}$**	1-454	H132A, N133A	6xHis-TEV	pET-30b	*S. pombe*
**Edc3$^{YjeF\text{-}mut}$**	1-454	K258E, N368A, K392E, W393A	6xHis-TEV	pET-30b	*S. pombe*
**Edc3$^{HN/YjeF\text{-}mut}$**	1-454	H132A, N133A, K258E, N368A, K392E, W393A	6xHis-TEV	pET-30b	*S. pombe*
**Not1^{MIF4G}**	829-1090	—	MBP-3C	pMAL	*S. pombe*

Table S2. *EDC3* constructs used in yeast growth assays

plasmid	mutations	epitope tag	vector	organism
EDC3^{WT}	—	13xMyc	YCplac33	*S. cerevisiae*
EDC3$^{HN\text{-}mut}$	H138A, N139A	13xMyc	YCplac33	*S. cerevisiae*
EDC3$^{YjeF\text{-}mut}$	G325Y, R331E, N336A	13xMyc	YCplac33	*S. cerevisiae*
EDC3$^{HN/YjeF\text{-}mut}$	H138A, N139A, G325Y, R331E, N336A	13xMyc	YCplac33	*S. cerevisiae*
EDC3^{ADA}	F105A, F107A	13xMyc	YCplac33	*S. cerevisiae*
EDC3$^{ADA/HN\text{-}mut}$	F105A, F107A, H138A, N139A	13xMyc	YCplac33	*S. cerevisiae*
EDC3$^{ADA/YjeF\text{-}mut}$	F105A, F107A, G325Y, R331E, N336A	13xMyc	YCplac33	*S. cerevisiae*
EDC3$^{ADA/HN/YjeF\text{-}mut}$	F105A, F107A, H138A, N139A, G325Y, R331E, N336A	13xMyc	YCplac33	*S. cerevisiae*

Table S3. Single-turnover ATPase rates of Dhh1 in the presence of wild-type and mutant Edc3 and Not1^{MIF4G}

	k_{obs} (h^{-1})	Figure(s)
**Dhh1**	0.034 ± 0.003	2b, 2e, 3d, S1b, S9a, S11b, S12a
**Dhh1 + Edc3**	0.157 ± 0.003	S1b, S11b, S12a
**Dhh1 + Edc3^{ADA}**	0.175 ± 0.008	2b
**Dhh1 + Edc3$^{\Delta Lsm}$**	0.151 ± 0.007	2e
**Dhh1 + Edc3$^{\Delta YjeF\text{-}N}$**	0.075 ± 0.008	2e
**Dhh1 + Edc3^{FDF}**	0.044 ± 0.004	2e
**Dhh1 + Edc3$^{HN\text{-}mut}$**	0.113 ± 0.006	3d
**Dhh1 + Edc3$^{YjeF\text{-}mut}$**	0.082 ± 0.005	3d
**Dhh1 + Edc3$^{HN/YjeF\text{-}mut}$**	0.064 ± 0.006	3d
**Dhh1 + Not1^{MIF4G}**	0.224 ± 0.009	S9b, S11b, S12a
**Dhh1 + Edc3 + Not1^{MIF4G}**	2.25 ± 0.08	S11b

Table S4. Multiple-turnover ATPase rates of Dhh1 in the presence of wild-type and mutant Edc3 and Not1^{MIF4G}

	V_0 (µM/min)	Figure(s)
**Dhh1**	0.0244 ± 0.0015	2b, 2f, 3e
**Dhh1**	0.017 ± 0.003	5b, S1d, S9d, S12b
**Dhh1 + Edc3**	0.448 ± 0.012	2b, 2f, 3e
**Dhh1 + Edc3**	0.404 ± 0.005	5b, S1d, S12b
**Dhh1 + Edc3^{ADA}**	0.119 ± 0.003	2b
**Dhh1 + Edc3$^{\Delta Lsm}$**	0.414 ± 0.009	2f
**Dhh1 + Edc3$^{\Delta YjeF\text{-}N}$**	0.220 ± 0.014	2f
**Dhh1 + Edc3^{FDF}**	0.0289 ± 0.0012	2f
**Dhh1 + Edc3$^{HN\text{-}mut}$**	0.286 ± 0.007	3e
**Dhh1 + Edc3$^{YjeF\text{-}mut}$**	0.167 ± 0.007	3e
**Dhh1 + Edc3$^{HN/YjeF\text{-}mut}$**	0.144 ± 0.004	3e
**Dhh1 + Not1^{MIF4G}**	0.87 ± 0.03	5b, S12b
**Dhh1 + Edc3 + Not1^{MIF4G}**	3.90 ± 0.11	5b

Table S5. Values for maximal rate (k_{max}) and concentration of half-maximal rate ($K_{1/2}$) from single-turnover ATPase concentration series

	k_{max} (h^{-1})	$K_{1/2}$ (μM)	Figure(s)
**Dhh1**	0.60 ± 0.05	90 ± 12	1b, 4b, 5d
**Dhh1 + Edc3**	0.81 ± 0.02	2.0 ± 0.2	1b, 5d
**Dhh1 + Not1^{MIF4G}**	4.7 ± 0.6	82 ± 15	4b, 5d
**Dhh1 + Edc3 + Not1^{MIF4G}**	25.5 ± 1.4	17 ± 2	5d

Table S6. Equilibrium dissociation constants (K_D) of Dhh1 for mant-ATP, in the absence and presence of Edc3

	K_D (μM)	Figure
**Dhh1**	287 ± 2	1d
**Dhh1 + Edc3**	24.7 ± 1.5	1d

Table S7. Equilibrium dissociation constants (K_D) of Dhh1 for U_{20} RNA in the presence of Edc3 and Not1^{MIF4G} in the absence of nucleotide, or with different nucleotides and nucleotide analogs

	nucleotide	K_D (μM)	Figure
**Dhh1**	—	7.0 ± 0.6	1e
	ATP	7.5 ± 0.6	
	ADP-BeF_x	7.5 ± 0.7	
	ADP-AlF_4	7.0 ± 0.6	
	ADP	9.1 ± 0.8	
**Dhh1 + Edc3**	—	6.6 ± 0.8	1e
	ATP	0.44 ± 0.07	
	ADP-BeF_x	0.059 ± 0.006	
	ADP-AlF_4	7.6 ± 1.4	
	ADP	7.0 ± 0.9	
**Dhh1 + Not1^{MIF4G}**	—	6.8 ± 0.9	S10c
	ATP	6.4 ± 0.5	
	ADP-BeF_x	7.7 ± 0.8	
	ADP-AlF_4	8.0 ± 1.0	
	ADP	6.7 ± 0.9	
**Dhh1 + Edc3 + Not1^{MIF4G}**	—	5.9 ± 0.5	S10c
	ATP	0.83 ± 0.08	
	ADP-BeF_x	0.037 ± 0.003	
	ADP-AlF_4	4.3 ± 0.4	
	ADP	5.3 ± 0.5	

Table S8. Equilibrium dissociation constants (K_D) of Dhh1 for U_{20} RNA in the presence of wild-type and mutant Edc3 (unless otherwise noted, all data are in the presence of ADP-BeF_x)

	K_D (μM)	Figure(s)
**Dhh1**	6.3 ± 0.4	2c, 2g, 3f, S15b
**Dhh1 + Edc3**	0.059 ± 0.006	2c, 2g, 3f, S15b
**Dhh1 + $Edc3^{ADA}$**	6.9 ± 0.5	2c
**Dhh1 + $Edc3^{\Delta Lsm}$**	0.061 ± 0.004	2g
**Dhh1 + $Edc3^{\Delta YjeF\text{-}N}$**	4.5 ± 0.3	2g, 3f
**Dhh1 + $Edc3^{FDF}$**	4.7 ± 0.3	2g
**Dhh1 + $Edc3^{HN\text{-}mut}$**	0.130 ± 0.009	3f
**Dhh1 + $Edc3^{YjeF\text{-}mut}$**	3.6 ± 0.2	3f
**Dhh1 + $Edc3^{HN/YjeF\text{-}mut}$**	4.7 ± 0.3	3f
**Dhh1 + Edc3** *(AMP-PNP)*	3.6 ± 0.3	S16b

Table S9. Experimental masses for individual proteins and protein complexes as determined by mass photometry

	experimental mass (kDa)	theoretical mass (kDa)	Figure(s)
**Dhh1**	49 ± 13	Dhh1: 54.9	2h, 5e
**Dhh1**	66 ±18	Dhh1: 54.9	S2b
**Edc3**	95 ± 13	Edc3: 49.4<br>Edc3 dimer: 98.7	2h
**Dhh1 + Edc3**	98 ± 18	Dhh1: 54.9<br>Edc3: 49.4<br>Dhh1-Edc3 heterodimer: 104.2	2h, 5e, S2a, S6b
**Dhh1 + Edc3**	99 ± 25	Dhh1: 54.9<br>Edc3: 49.4<br>Dhh1-Edc3 heterodimer: 104.2	S2b
**Dhh1 + Edc3**	99 ± 18	Dhh1: 54.9<br>Edc3: 49.4<br>Dhh1-Edc3 heterodimer: 104.2	S14a
**Dhh1 + Edc3**<br>(+ glutaraldehyde)	97 ± 18	Dhh1: 54.9<br>Edc3: 49.4<br>Dhh1-Edc3 heterodimer: 104.2	S14a
**Dhh1 + Edc3^{ADA}** (150 mM NaCl)	97 ± 20	Dhh1: 54.9<br>Edc3^{ADA}: 49.2<br>Dhh1-Edc3^{ADA} heterodimer: 104.0	S2a
**Dhh1 + Edc3^{WT}** (500 mM NaCl)	101 ± 30	Dhh1: 54.9<br>Edc3: 49.4<br>Dhh1-Edc3 heterodimer: 104.2	S2a
**Dhh1 + Edc3^{ADA}** (500 mM NaCl)	67 ± 17<br>86 ± 24	Dhh1: 54.9<br>Edc3^{ADA}: 49.2<br>Edc3^{ADA} homodimer: 98.3<br>Dhh1-Edc3^{ADA} heterodimer: 104.0	S2a
**Dhh1 + Edc3$^{\Delta YjeF\text{-}N}$**	73 ± 17	Dhh1: 54.9<br>Edc3$^{\Delta YjeF\text{-}N}$: 21.3<br>Dhh1-Edc3$^{\Delta YjeF\text{-}N}$ heterodimer: 76.2	S2b
**Dhh1 + Edc3$^{HN\text{-}mut}$**	98 ± 18	Dhh1: 54.9<br>Edc3$^{HN\text{-}mut}$: 49.3<br>Dhh1-Edc3$^{HN\text{-}mut}$ heterodimer: 104.1	S6b
**Dhh1 + Edc3$^{YjeF\text{-}mut}$**	103 ± 2	Dhh1: 54.9<br>Edc3$^{YjeF\text{-}mut}$: 49.2<br>Dhh1-Edc3$^{YjeF\text{-}mut}$ heterodimer: 104.1	S6b
**Dhh1 + Edc3$^{HN/YjeF\text{-}mut}$**	103 ± 24	Dhh1: 54.9<br>Edc3$^{YjeF\text{-}mut}$: 49.1<br>Dhh1-Edc3$^{YjeF\text{-}mut}$ heterodimer: 104.0	S6b
**Dhh1 + Not1^{MIF4G}**	89 ± 14	Dhh1: 54.9<br>Not1^{MIF4G}: 30.3<br>Dhh1-Not1^{MIF4G} heterodimer: 85.2	5e

**Dhh1 + Not1^{MIF4G}**	90 ± 13	Dhh1: 54.9<br>Not1^{MIF4G}: 30.3<br>Dhh1-Not1^{MIF4G} heterodimer: 85.2	S14b
**Dhh1 + Not1^{MIF4G}**<br>(+ glutaraldehyde)	88 ± 12	Dhh1: 54.9<br>Not1^{MIF4G}: 30.3<br>Dhh1-Not1^{MIF4G} heterodimer: 85.2	S14b
**Edc3 + Not1^{MIF4G}**	96 ± 14	Edc3: 49.4<br>Edc3 homodimer: 98.7<br>Not1^{MIF4G}: 30.3	S14c
**Edc3 + Not1^{MIF4G}**<br>(+ glutaraldehyde)	98 ± 20	Edc3: 49.4<br>Edc3 homodimer: 98.7<br>Not1^{MIF4G}: 30.3	S14c
**Dhh1 + Edc3 + Not1^{MIF4G}**	95 ± 22	Dhh1: 54.9<br>Edc3: 49.4<br>Not1^{MIF4G}: 30.3<br>Dhh1-Edc3 heterodimer: 104.2<br>Dhh1-Edc3- Not1^{MIF4G}: heterotrimer: 134.5	5e
**Dhh1 + Edc3 + Not1^{MIF4G}**	95 ± 17	Dhh1: 54.9<br>Edc3: 49.4<br>Not1^{MIF4G}: 30.3<br>Dhh1-Edc3 heterodimer: 104.2<br>Dhh1-Edc3- Not1^{MIF4G}: heterotrimer: 134.5	S14d
**Dhh1 + Edc3 + Not1^{MIF4G}**<br>(+ glutaraldehyde)	127 ± 26	Dhh1: 54.9<br>Edc3: 49.4<br>Not1^{MIF4G}: 30.3<br>Dhh1-Edc3 heterodimer: 104.2<br>Dhh1-Edc3- Not1^{MIF4G}: heterotrimer: 134.5	5e
**Dhh1 + Edc3 + Not1^{MIF4G}**<br>(+ glutaraldehyde)	118 ± 27	Dhh1: 54.9<br>Edc3: 49.4<br>Not1^{MIF4G}: 30.3<br>Dhh1-Edc3 heterodimer: 104.2<br>Dhh1-Edc3- Not1^{MIF4G}: heterotrimer: 134.5	S14d

Table S10. Single-turnover ATPase rates of Dhh1^{WT} and Dhh1^{DQAD} in the presence of Edc3 and Not1^{MIF4G} in either the presence or absence of RNA

			**k_{obs} (h^{-1})**	**Figure**
**Dhh1**	Dhh1^{WT}	+RNA	0.036 ± 0.003	4c
		–RNA	0.033 ± 0.003	4c
	Dhh1^{DQAD}	+RNA	0.035 ± 0.003	4c
		–RNA	0.033 ± 0.002	4c
**Dhh1 + Not1^{MIF4G}**	Dhh1^{WT}	+RNA	0.188 ± 0.009	4c
		–RNA	0.0339 ± 0.0016	4c
	Dhh1^{DQAD}	+RNA	0.039 ± 0.003	4c
		–RNA	0.0344 ± 0.0019	4c
**Dhh1 + Edc3**	Dhh1^{WT}	+RNA	0.136 ± 0.004	4c
		–RNA	0.128 ± 0.005	4c
	Dhh1^{DQAD}	+RNA	0.143 ± 0.004	4c
		–RNA	0.132 ± 0.004	4c
**Dhh1 + Edc3 + Not1^{MIF4G}**	Dhh1^{WT}	+RNA	2.25 ± 0.08	S12a
		–RNA	0.136 ± 0.003	S12a
	Dhh1^{DQAD}	+RNA	0.148 ± 0.004	S12a
		–RNA	0.139 ± 0.005	S12a

Table S11. Multiple-turnover ATPase rates of Dhh1^{WT} and Dhh1^{DQAD} in the presence of Edc3 and Not1^{MIF4G} in either the presence or absence of RNA

			V_0 (μM/min)	Figure(s)
**Dhh1**	Dhh1^{WT}	+RNA	0.017 ± 0.003	4d
		–RNA	0.0015 ± 0.0003	4d
	Dhh1^{DQAD}	+RNA	0.0042 ± 0.0011	4d
		–RNA	0.0017 ± 0.0003	4d
**Dhh1 + Not1^{MIF4G}**	Dhh1^{WT}	+RNA	0.87 ± 0.03	4d
		–RNA	0.044 ± 0.002	4d
	Dhh1^{DQAD}	+RNA	0.043 ± 0.003	4d
		–RNA	0.032 ± 0.002	4d
**Dhh1 + Edc3**	Dhh1^{WT}	+RNA	0.404 ± 0.005	4d
		–RNA	0.248 ± 0.005	4d
	Dhh1^{DQAD}	+RNA	0.051 ± 0.003	4d
		–RNA	0.040 ± 0.002	4d
**Dhh1 + Edc3 + Not1^{MIF4G}**	Dhh1^{WT}	+RNA	3.89 ± 0.11	S12b
		–RNA	0.458 ± 0.017	S12b
	Dhh1^{DQAD}	+RNA	0.091 ± 0.006	S12b
		–RNA	0.079 ± 0.004	S12b

Table S12. Western blot quantification of Myc-tagged Edc3, normalized to GAPDH loading control (related to Fig. S8)

	Myc/GAPDH
**Edc3^{WT}**	0.83 ± 0.16
**Edc3$^{HN\text{-}mut}$**	1.0 ± 0.3
**Edc3$^{YjeF\text{-}mut}$**	1.0 ± 0.3
**Edc3$^{HN/YjeF\text{-}mut}$**	1.1 ± 0.3
**Edc3^{ADA}**	2.1 ± 0.6
**Edc3$^{ADA/HN\text{-}mut}$**	2.0 ± 0.5
**Edc3$^{ADA/YjeF\text{-}mut}$**	1.7 ± 0.4
**Edc3$^{ADA/HN/YjeF\text{-}mut}$**	1.9 ± 0.5

SUPPLEMENTAL METHODS

Sequence Conservation Analysis

Sequence conservation of Edc3 and Scd6 was generated using ConSurf (2); per-residue conservation was visualized using Seq2Logo (3).

Western Blotting

Samples from *scd6Δedc3Δ* transformants used in cell growth spotting assays were prepared by trichloroacetic acid extraction as previously described (4), and immunoblot analysis was performed as previously described (5). Steady-state Edc3 expression was probed using anti-Myc antibody with GAPDH used as a loading control. Densitometric analysis of western blot bands was performed using ImageJ. The signal intensity of each Myc-tagged protein was formalized to its respective GAPDH loading control.

SUPPLEMENTAL REFERENCES

1. Erdős,G. and Dosztányi,Z. (2024) AIUPred: combining energy estimation with deep learning for the enhanced prediction of protein disorder. *Nucleic Acids Research*, **52**, W176–W181.

2. Ben Chorin,A., Masrati,G., Kessel,A., Narunsky,A., Sprinzak,J., Lahav,S., Ashkenazy,H. and Ben-Tal,N. (2020) ConSurf-DB: An accessible repository for the evolutionary conservation patterns of the majority of PDB proteins. *Protein Sci*, **29**, 258–267.

3. Thomsen,M.C.F. and Nielsen,M. (2012) Seq2Logo: a method for construction and visualization of amino acid binding motifs and sequence profiles including sequence weighting, pseudo counts and two-sided representation of amino acid enrichment and depletion. *Nucleic Acids Research*, **40**, W281–W287.

4. Reid,G.A. and Schatz,G. (1982) Import of proteins into mitochondria. Yeast cells grown in the presence of carbonyl cyanide m-chlorophenylhydrazone accumulate massive amounts of some mitochondrial precursor polypeptides. *J Biol Chem*, **257**, 13056–13061.

5. Nanda,J.S., Cheung,Y.-N., Takacs,J.E., Martin-Marcos,P., Saini,A.K., Hinnebusch,A.G. and Lorsch,J.R. (2009) eIF1 Controls Multiple Steps in Start Codon Recognition during Eukaryotic Translation Initiation. *Journal of Molecular Biology*, **394**, 268–285.
